# Signature Optical Cues: Emerging Technologies for Monitoring Plant Health

**DOI:** 10.3390/s8053205

**Published:** 2008-05-16

**Authors:** Oi Wah Liew, Pek Ching Jenny Chong, Bingqing Li, Anand K. Asundi

**Affiliations:** 1 Centre for Biomedical and Life Sciences, Singapore Polytechnic, 500 Dover Road, Singapore 139651; 2 School of Mechanical and Aerospace Engineering, Nanyang Technological University, 50 Nanyang Avenue, Singapore 639798

**Keywords:** Thermography, fluorescence, leaf reflectance, plant stress, CIE color space, red edge, phytosensors, transgenic plants, reporter genes, inducible promoters, remote sensing

## Abstract

Optical technologies can be developed as practical tools for monitoring plant health by providing unique spectral signatures that can be related to specific plant stresses. Signatures from thermal and fluorescence imaging have been used successfully to track pathogen invasion before visual symptoms are observed. Another approach for non-invasive plant health monitoring involves elucidating the manner with which light interacts with the plant leaf and being able to identify changes in spectral characteristics in response to specific stresses. To achieve this, an important step is to understand the biochemical and anatomical features governing leaf reflectance, transmission and absorption. Many studies have opened up possibilities that subtle changes in leaf reflectance spectra can be analyzed in a plethora of ways for discriminating nutrient and water stress, but with limited success. There has also been interest in developing transgenic phytosensors to elucidate plant status in relation to environmental conditions. This approach involves unambiguous signal creation whereby genetic modification to generate reporter plants has resulted in distinct optical signals emitted in response to specific stressors. Most of these studies are limited to laboratory or controlled greenhouse environments at leaf level. The practical translation of spectral cues for application under field conditions at canopy and regional levels by remote aerial sensing remains a challenge. The movement towards technology development is well exemplified by the Controlled Ecological Life Support System under development by NASA which brings together technologies for monitoring plant status concomitantly with instrumentation for environmental monitoring and feedback control.

## 1. Introduction

With mounting pressures on ensuring food security while balancing resource utilization and environmental quality, the quest for practical tools to provide cues to plant stresses has received increased impetus [1]. Much effort has been geared towards developing strategies for non-destructive, pre-visual detection (and, if possible, quantification of the severity) of abiotic plant stresses to facilitate timely delivery of appropriate amounts of resource inputs, for example, water and nutrients. A vast number of studies have enhanced our understanding of the optical properties of leaves and their correlation with plant responses to various stresses. Infrared/near infrared analyses, thermography, chlorophyll fluorescence analyses and transmission/reflectance spectral indices have been used to monitor water status, surface temperature, photosynthetic efficiency and structural changes in plants for early detection of environmental stress responses [2]. Recent studies have shown that it is possible to tease out signature spectral changes that are diagnostic of specific deviations in plant health. There are two broad ways to achieve this: 1) by capturing the spectrum directly from a plant surface and identifying unique spectral features that change in response to stress; 2) by signal creation whereby plants are endowed by genetic engineering to emit specific optical cues in response to stress. In the latter approach, genetically modified plants harboring optically active reporters under the control of inducible promoters have shown promise as phytosensors of stress situations. These technologies when sufficiently developed for large scale field applications serve to drive sustainability in agriculture towards reality.

It is not difficult to envisage that with broad climatic changes on a global scale, a growing world population and rapidly declining arable land, it may become necessary to move crop production from the terrestrial to extraterrestrial realm to meet escalating food demands. Even now, a futuristic extraterrestrial extension of crop production driven by the National Aeronautics and Space Administration's (NASA's) concept of Advance Life Support (ALS) has become a research priority with increasing recognition that plants are key “engines” of a self-sustaining system for cycling air, water, nutrients and wastes in a controlled environment for long term space habitation [3]. To spearhead these efforts, elucidation of signature spectral cues that reflect the health status of plants in simulated ground-based and spaceflight experiments are pivotal to resolving plant responses and adaptations to extraterrestrial environments. The integration of optical monitoring of plant spectral characteristics with feedback control of atmospheric composition, water, nutrients and temperature would be instrumental to the successful development of life support systems in hostile spaceflight environments.

This paper reviews strategies used to identify signature spectral features and correlate these with specific plant stresses. It highlights the difficulties imposed by limited understanding of the regulatory networks involved in plant responses and adaptations to stress although the fundamental concepts have become better resolved in the past decade. This paper also discusses aspects of optical instrumentation that are critical to the development of sensitive and robust monitoring systems as well as opportunities for remote sensing. These systems must also be integrated with appropriate strategies for spectral analyses that are consistent with basic plant processes.

## 2. Stress-Associated Leaf Spectral Properties

### 2.1 Fundamentals of plant stress sensing

Leaf optical responses to a wide range of biotic and abiotic stresses have been widely researched [2, 4-5]. These include responses to increased CO_2_ and other gaseous pollutants [6-7], heat stress [8-9], heavy metal toxicity [10], exposure to ultraviolet radiation [11], water status [8, 12], insect pest attack [13], herbicide treatment [14], salinity effects [15] and extremes in nutrient availability [16]. In many studies, the spectral wavebands investigated as predictors of plant health status across species range from 400 – 2500 nm [5]. The logic behind these correlations is that unfavorable growing conditions result in morphological, physiological and/or biochemical changes that impact on the manner with which plants interact with light. Reflectance characteristics in the 400 – 700 nm range are primarily influenced by the cellular level of colored pigments like chlorophyll, anthocyanins and carotenoids [17-18], in the 700 – 1400 nm range by cell structure [19] and in the 1400 – 2000 nm range by the water content in the tissues [20]. Leaf reflectance patterns have been employed to measure leaf chlorophyll content [21-23], N status [24-25], xanthophylls and carotenoid pigment levels [26-27].

### 2.2 Techniques for measuring plant stress

#### 2.2.1 Thermography and Fluorescence

Perturbations to the processes of transpiration and photosynthesis can be exploited as cues for plant stresses. Control of transpirational water loss through stomatal openings on plant leaves constitutes an important mechanism for maintaining leaf surface temperature. In the event of water stress, decreased transpirational cooling from stomatal closure leads to an increase in leaf temperature that could be monitored by thermography [28-30]. Thermal imaging combined with extraction of additional information from visible imaging has been described as an improved technique for correlating plant surface temperature variation to stomatal conductance and diagnosis of water deficit stress at canopy level [31-32]. Biotic stresses are also detectable by thermography since pathogen-mediated increase in a central plant defense compound, salicylic acid, results in stomatal closure and a concomitant increase in temperature. This series of events has been exploited to allow early detection of tobacco virus infection by thermography [33]. The thermal effect resulting from plant-pathogen interaction has allowed tracking of disease progression even at the early presymptomatic stage under controlled environmental conditions [34].

Chlorophyll a is the predominant pigment contributing to red fluorescence in leaves while chlorophyll b constitutes an accessory pigment accounting for about one-third (or less) of total leaf chlorophyll content. Excess energy from light harvested by chlorophylls or transferred to chlorophylls by other accessory pigments (carotenoids and anthocyanins) and not utilized in the photosynthetic transport chain may be dissipated as heat or expended into lifting chlorophyll chromophores from ground-state to high-energy states. De-excitation via emission of photons at a longer wavelength leads to red fluorescence. Plant stresses that impair photosynthesis lead to greater accumulation of excess light energy dissipated as chlorophyll fluorescence. The negative correlation between *in vivo* chlorophyll fluorescence and photosynthesis has been the focus of numerous studies correlating various chlorophyll fluorescence signatures with plant stresses [35-37].

In fluorescence sensing, excitation of green leaves with UV-A (Ex 400 nm) or blue light (Ex 470 nm), give rise to red and far red chlorophyll a fluorescence emissions around 690 nm and 740 nm [38]. Fluorescence intensity ratios in these red and far red wavebands (F690/F740) have been used as indicators of physiological strain but because many natural and stress factors impact on chlorophyll fluorescence, identification of specific stressors is not possible. For example, F690/F730 has been shown to increase under nitrogen, phosphorus and potassium deficiency in sunflower [35] and water deficit in poplars and conifers [37, 39]. Simultaneous thermal and fluorescence imaging constitutes a multispectral approach for characterization of plant stresses [40-41].

#### 2.2.2 Leaf Reflectance

Leaf spectral reflectance provides a vast data resource for assessing plant health based on the impact of biotic and abiotic stresses on leaf biochemistry and anatomy which in turn produces distinct changes in leaf optical properties. Key regions of a reflectance spectrum are:

1. blue region (400 – 499 nm) which is strongly influenced by absorption of chlorophylls and carotenoids.
2. blue-green edge (500 – 549 nm) leading to the green peak at 550 nm.
3. red edge (650 – 699 nm) associated with strong chlorophyll absorption.

#### 2.2.3 Biochemical basis for leaf reflectance properties

Light falling on a leaf can be reflected, absorbed or transmitted. Absorption in the visible (VIS) and infrared (IR) regions of the spectrum is primarily driven by stretching and bending of covalent bonds between oxygen, carbon, hydrogen and nitrogen present in plant biochemical components like sugar, lignin, cellulose and proteins [42]. In addition, pigments responsible for leaf color also constitute principal absorbing molecules. A large number of natural pigments found in plants comprise closed ring tetrapyrroles with centrally complexed metals. The range of colors is derived primarily from the structures of the tetrapyrrole rings and peripheral substitutions rather than the bound metal [43]. The predominant green chlorophylls found in photosynthetic tissues of higher plants are reduced porphyrins (dihydrophorphyrins) containing a centrally bound Mg^2+^ ion and linked to a long hydrophobic phytol tail through esterification of the acid group at C-17 (IUPAC-IUB nomenclature) [44]. The absorption spectrum of intact chlorophyll molecules shows two dominant bands in the red (Q band) and blue (Soret band) regions and an absorption minimum around 550 nm, giving rise to the perception of a green color. The presence of a methyl group in chlorophyll a instead of an aldehyde group in chlorophyll b at C-7 position accounts for differences in the absorption wavelengths of the Q (669 nm vs 644 nm) and Soret (432 nm vs 455 nm) bands. Because of the central function of these pigments in photosynthesis, chlorophyll content is generally regarded as a good indicator of plant physiological health [45]. Many nutrient deficiencies result in a decrease in chlorophyll content, a concomitant increase in reflectance in the visible (400 – 700 nm) and infrared (700-1100 nm) ranges and blue shift in the red edge inflection point [46-47]. Visually, chlorotic changes are perceived as yellowing of leaves [48].

#### 2.2.4 Anatomical basis for leaf reflectance properties

**Figure 1.**
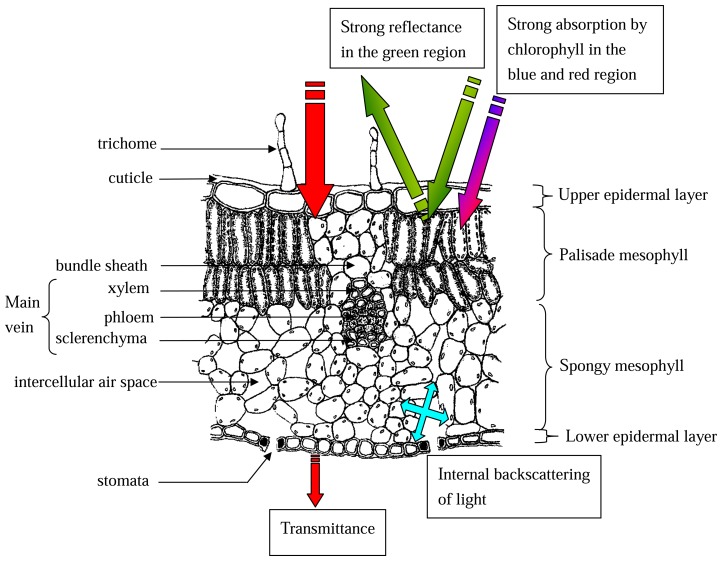
Schematic representation of a vertical section through a typical leaf including the main vein and surrounding tissues. The interaction of light with various cellular layers in relation to anatomical and biochemical characteristics are highlighted. The vascular tissue of the main vein is delineated from the spongy mesophyll by a compact layer of parenchyma cells called the bundle sheath. Sheath cells may or may not contain a few chloroplasts. Enclosed within the bundle sheath are adaxial xylem, abaxial phloem and some supporting sclerenchyma cells. A network of connecting vascular strands forms a continuous system throughout the leaf with branches of increasingly finer dimensions originating from the main vein. Point spectral data collection over leaf and main veins should be avoided to reduce variations in spectral measurements [87].

Reflectance patterns are influenced by leaf surface features, internal architecture and biochemical composition. Figure 1 shows a schematic representation of the key anatomical structures in relation to their mode of interaction with light. A dorsiventral leaf is bounded externally by an upper and lower epidermis. Epidermal cells vary widely in cell structure and are closely knit together with no spaces between them except at stomatal pores. Stomata perforate both epidermal layers but particularly on the lower abaxial side. Unlike the guard cells that surround each stomatal pore, epidermal cells do not contain chloroplasts. Convexity of the epidermal cells can act to collect and focus light, thus increasing the propensity for chlorophyll pigment found in the deeper cell layers to intercept photons of light [49-50]. Epidermal cell walls are characteristically impregnated with a waxy substance, cutin, that also forms an outer superficial layer called the cuticle, especially on the upper adaxial surface. The surface texture of the cuticle may be smooth, spiny, ridged or cracked depending on species. The cuticle varies in thickness according to species and environmental conditions. Cuticle thickness significantly influences the amount of light reflected at an angle complementary to the angle of incidence, affecting the reception and redistribution of light at the air-cuticle interface [17, 51-52]. Changes to cuticular thickness can alter leaf surface reflectance patterns. Slaton et al. (2001) [19] found that cuticle thickness greater than 1 μm constitute one of the key predictors of near infrared (NIR) leaf reflectance. Unicellular or multicellular epidermal appendages called trichomes may be present on both surfaces. Trichome architecture varies widely according to species. Trichome density exhibits spatiotemporal variation and is positively correlated with ambient temperature, inversely correlated with nutrient status and negatively correlated with leaf water potential. High trichome density can distort reflectance spectra in the visible light range. Trichomes or hairs greatly enhance surface reflectance in the visible region but their influence in the NIR region is variable [19, 53-54].

The palisade mesophyll is composed of specialised parenchyma cells elongated in a direction perpendicular to the leaf surface. These cells also contain numerous discoid chloroplasts that absorb strongly in the red and blue region. Light that is collected and focused by convex epidermal cells are transmitted to the tube-like palisade cells. These cells act as light conduits that propagate visible light further into the internal tissue layers. Below the palisade layer is the spongy mesophyll which is composed of loosely arranged spherical or irregularly shaped cells containing fewer chloroplasts. A characteristic of the spongy mesophyll is the interconnecting air spaces that form a continuum void area. The shape of the palisade and spongy mesophyll cells (long cylindrical versus spherical), the thickness of the spongy mesophyll layer and the ratio of mesophyll cell surface area exposed to the intercellular air spaces expressed per unit leaf area can influence the penetration of light within the leaf, the optical path length and the degree of light backscattering [18, 55-57]. Furthermore, reflectance patterns in the NIR region (700 -1300 nm) have been attributed to the air-cell interfaces within the spongy mesophyll. This effect is due to differences in the refractive indices between the hydrated cell walls and the intercellular air spaces resulting in backscattering of light and weak absorptance at the NIR region [58-59]. Currently, there is still a lack in clarity of the combined contributions of each anatomical feature to tissue optical properties. The degree of hydration of a leaf also influences its spectral properties whereby reflectance of a dry leaf is higher than that of fresh leaves across the visible range. Absorption of water determines the shape of the middle infrared reflectance curve with strong absorption bands around wavelengths at 1400 nm and 1900 nm.

Models varying in complexity have been developed to explain the propagation of light through a leaf. An early “plate model” described by Allen et al. (1969) [60] considered a typical leaf as a unique compact plate specified by two constants: an effective refractive index and an effective absorption coefficient. Further improvements to this model were made by accounting for the scattering of light in the void area of the leaf, water content and the interaction of light across dissimilar layers: 2 cuticle and epidermal layers, pigment-enriched palisade layer and spongy mesophyll [61-64]. So far, reliable and accurate quantitative models that relate leaf reflectance in the visible and NIR region to structural and biochemical characteristics remain elusive. The extent to which these relationships can be generalized across species, leaf developmental stages, growing conditions and environmental factors remains in question.

#### 2.2.5 Vegetative indices derived from leaf reflectance spectra

Spectral indices that are good predictors of leaf pigment content have been established for many crop types [65]. While chlorophyll content can be estimated from equations derived on the basis of their absorption characteristics [66], various spectral indices based on reflectance spectroscopy have also been developed. The latter methods are advantageous in that they do not require destructive sampling for measurements and thus facilitate monitoring of pigment changes over time. Early studies have indicated that the refractive index of internal wet cell walls and internal backscattering is invariant with wavelength in the VIS-IR region [59]. Thus, strategies in the development of spectral indices commonly use reflectance ratios derived from dividing leaf reflectance at stress-sensitive wavelengths by that at stress-insensitive wavelength. This effectively cancels out the effects caused by internal reflections and hence provides stronger quantitative relationships with chlorophyll content [14]. Equations that employ ratios of leaf reflectance at different wavelengths in the visible and near infrared regions to estimate leaf pigment content include Simple Ratio (SR) [67], Normalized Difference (ND) [65], Plant Senescence Reflectance Index (PSRI) [68] and Photochemical Reflectance Index (PRI) [69]. Refinement of SR and ND indices by incorporating a waveband in the blue region to correct for external leaf reflectance improved their predictive accuracy of leaf chlorophyll concentrations [65, 70]. Other dimensionless spectral indices have also been derived from second derivative calculations of the reflectance spectrum. An example is the Yellowness Index (YI) that estimates the degree of leaf chlorosis on the basis of the concavity-convexity of the reflectance spectrum at the central wavelength between the reflectance maximum and minimum at 550 nm and 670 nm, respectively [48].

**Figure 2.**
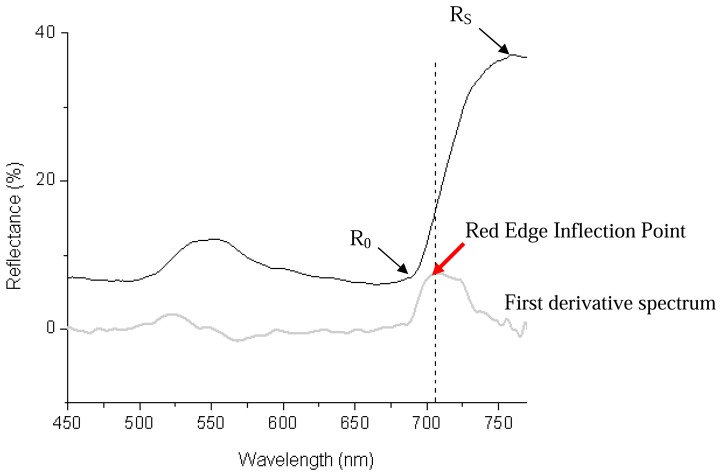
Typical leaf reflectance spectrum across the visible and near infrared region and its first derivative (adapted from Li et al. 2005 [78]).

Red edge has been employed as a vegetative index of choice for determination of abiotic and biotic plant stresses. This parameter is a consequence of strong absorption by chlorophyll in the red region coupled with strong reflectance in the infrared region arising from internal light scattering in the leaf and lack of pigment absorption at wavelengths greater than 700 nm (Figure 2). Thus, red edge represents the point of maximum slope between the low reflectance red region (∼680 nm) arising from chlorophyll absorption and the high reflectance infrared region (∼750 nm) attributed to internal scattering within the leaf [71].

The red edge inflection point (REIP) can be determined by one of four methods. The simplest is by linear interpolation where REIP is estimated from the slope of the straight line centered between two reference points: the shoulder reflectance maximum R_S_ and reflectance minimum R_0_ [72]. A second method estimates REIP as the mid-point of the ascending edge of a fitted normal curve to the reflectance red edge derived by an inverted Gaussian technique [73]. The weakness of these first two methods is their dependence on two or more reference points and poor resolution of interfering background absorption. A third method employing high-order curve fitting techniques locates REIP as the maximum first derivative of the reflectance spectrum [74-76]. The first derivative of a typical reflectance spectrum is depicted in Figure 2 (grey line). However, the accuracy of REIP estimates is highly dependent on the continuity and spectral resolution of the detecting sensor. To overcome this disadvantage, a fourth technique based on a three-waveband Lagrangian interpolation technique that uses a second polynomial fitting procedure on the first-order derivative spectrum was proposed by Dawson and Curran (1998) [77].

Shifts in the red edge position can be a robust and sensitive predictor of plant stress by virtue of reduced absorption from falling levels of chlorophyll and a decrease in infrared reflectance due to changes in plant cell structure [78]. Red edge is known to be relatively unaffected by high trichome density, leaf structural variations [65, 79] and leaf chlorophyll heterogeneity [80], and is the index of choice where leaf anatomical changes are suspected to occur concurrently with specific stresses or environmental conditions. In many studies, it has been observed that as healthy plants progress towards maturity, the red edge position shifts to longer wavelengths stabilizing around 712 – 715 nm. On the other hand, the red edge position in stressed plants is often at shorter wavelength positions compared with normal [78, 81-82]. Thus, plant stresses appear to be associated with an inhibition of normal shifts in the red edge position towards longer wavelengths.

**Figure 3.**
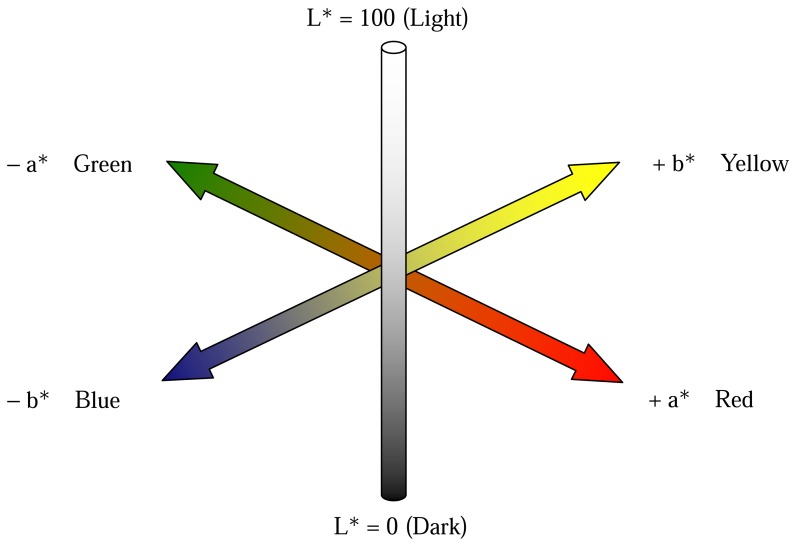
Three-dimensional CIELAB color space (adapted from Li et al. 2005 [96]).

A different approach in analyzing leaf reflectance spectrum employs a color description system that models color perception over the entire visible range. Since many plant stress factors impact on leaf biochemistry and morphology and consequently on reflectance spectral characteristics in the visible range, it follows that these changes can be related to leaf color. Hence, an analysis of stress in terms of leaf colorimetric response represents an entirely valid option [83]. The CIE color space defined by the Commission International de l'Eclairage in 1976 [84], known as CIE 1976 L*a*b* (Figure 3), provides a three dimensional representation for the perception of color stimuli to a standard observer under strictly standardized light sources.

The central vertical axis of the color space represents the L* or lightness coordinate which has a value from 0 (black) to 100 (white). The two chroma coordinates, a* and b*, represent red/green and yellow/blue color, respectively. The 0 value for both a-a′ and b-b′ axes represent neutral grey. If two points in the color space, representing two stimuli, are coincident, then there is no color difference between the two stimuli. As the perceived color difference between the two stimuli increases, the distance in the color space between these two points increases accordingly. One measure of the difference in color between two stimuli is the Euclidean distance, ΔE*, the derivation of which is described by Wyszecki and Stiles (2000) [85].

### 2.3 Challenges to teasing out signature optical stress cues

#### 2.3.1 Confounding factors and limitations in obtaining signature cues for specific stresses

Price (1994) [86] alluded to the difficulty in defining unique spectral signatures in plants despite advances in optical technologies. The dynamic nature of leaf reflectance properties as a function of natural cycles of leaf flush and senescence, diurnal cycles and environmental factors such as light and nutrient availability can result in significant within-species or even within-plant variability leading to confusion over spectral recognition of stress responses. In terms of nutrient stress, one significant confounding factor is that deficiency of one element can result in secondary deficiency in other essential elements. Under prolonged experimental deprivation of boron, the plant's ability to accumulate calcium is impaired. Calcium deficiency can in turn lead to potassium deficiency. Thus, signature spectral cues must be derived at early stages of deficiency to obviate difficulties in data interpretation when two or more essential elements become deficient at the same time. Another confounding factor is the difference in response depending on environmental growth conditions. Although the pattern of reflectance changes in response to various nutrient deficiencies is the same under greenhouse and controlled growth chamber conditions, the level of deviation under controlled growth conditions is lesser [47]. Furthermore, the influence of variations in cuticular thickness and leaf trichome density and their combined effects on reflectance patterns [51, 79] are not clearly understood. Baltzer and Thomas (2005) [17] found that unstressed plants exposed to moderate spatial and temporal variations in nutrient and light availability typical of that in natural vegetated areas display changes in spectral reflectance patterns in the visible region similar to that produced by acute plant stress. Thus, use of broad changes in reflectance spectra in terms of shape and amplitude in the visible light and NIR region will not necessarily be reflective of status of plant health. These spectral parameters cannot provide definitive information on the absence or presence of specific stresses because of substantial inherent variations within-leaf, within-plant and within-species across growing sites and seasons [87]. Thus, thorough characterization of natural variations in leaf optical properties within species under normal growing conditions is critical and this includes:

1) Within-leaf: Multiple measurements per individual leaf at various positions; basal, middle or leaf tip and margins, leaf blade versus main vein. Castro-Esau et al. (2006) [87] reported greater variation in spectral measurements taken over the leaf blade particularly over leaf veins and took precautions to avoid the main vein. This is not surprising since chlorophyll is not distributed evenly across the leaf blade [87-88].
2) Within-plant: Multiple leaves sampled per plant at terminal young, middle or older basal positions. In many plant species, the terminal young leaves are generally of a lighter green shade compared with the more mature regions.
3) Between plants within species and same growing conditions: Measurements from multiple plants growing in the same site using the same spectrometer and collection parameters.

The key to classification of plant health status lies in the creative combination of a set of highly discriminating features from the spectral data and development of powerful pattern recognition tools, along with in-depth knowledge of target plant biochemistry, anatomy and phenology for successful discrimination of stress responses against normal variability. In addition, the use of a spectrometer with a greater range (350 nm up to 2500 nm) and a higher resolution (<0.5 nm) could provide better potential for teasing out discriminatory spectral features in relation to stress responses.

#### 2.3.2 Strategies in teasing out signature stress cues

Changes in leaf spectral characteristics have been exploited for correlation with plant nutrient deficiencies. Ayala-Silva and Beyl (2005) [47] reported an increase in reflectance in the VIS-IR range especially in the region from 675 – 755 nm for plants deficient in N, P, K, Ca and Mg while Ponzoni and Goncalves (1999) [89] observed decreased reflectance values for P- and K-deficient plants. Other workers have reported marked increase in reflectance in the 650 – 1100 nm region as being associated with total chlorophyll [23] and water content [90]. Rises in reflectance is well correlated with deficiencies associated with leaf chlorosis and senescence. Graeff and Claupein (2003) [91] evaluated leaf reflectance scans over prescribed ranges in the visible and near infrared regions within the L*a*b* color system (CIE, 1976) for distinguishing nitrogen deficiency in the field. It was found that the b* values within the 516-780 nm wavelength range increased significantly compared with N-sufficient plants. However, since b* decreased in both N-sufficient and N-deficient plants as a function of crop age, deficiency could not be defined by an absolute b* value. Thus, the color distance in the b* parameter between N-sufficient and N-deficient plants, ΔE_b_, that mathematically eliminates the contribution of other plant factors on the b* parameter was more suitable as an indicator of N status.

Many studies have predominantly focused on the general effects of one or two plant stresses on leaf spectral characteristics or how several different stresses lead to changes in one or two spectral features. For example, deficiencies in nitrogen, phosphorus, potassium, magnesium, calcium and iron in different crop types are all associated with blue-shifts of varying magnitudes in the red-edge position [46-47, 78, 91-92]. Thus, tracking shifts in the red edge position alone does not provide discriminatory information on specific stresses. In order for early and reliable diagnosis of specific elemental deficiencies, ideally a combinatorial change in spectral parameters that is unique to each kind of plant stress should be identified.

To a limited extent, Adams et al. (2000a and b) [93-94] was able to use a discriminant analysis method with four spectral predictors (*F_o_/F_v_*: ratio of minimal fluorescence to variable fluorescence; *F_o_/F_5min_*: ratio of minimal fluorescence to the fluorescence yield after 5 min illumination; YI: Yellowness Index; NDVI: Normalized Difference Vegetation Index) to distinguish Cu, Fe, Mn and Zn deficiencies in soybean. Graeff and Claupein (2007) [12] described the use of the L*a*b* color system with a* and b* parameters determined between selected wavelength ranges of 510 – 780 nm, 540 – 780 nm, 490 – 1300 nm and 540 – 1300 nm for successful discrimination of nutrient stress from water stress. The color parameter b* was found to increase with severity of water stress, a spectral change that could also be indicative of N deficiency alluded to in their earlier work. Using an additional parameter, total color difference between stressed and unstressed plants (ΔEab), discrimination of water from nutrient stress was achieved. While ΔEab remains unchanged under water stress conditions, this value increased significantly with increasing severity of N, P, Mg and Fe deficiency.

**Figure 4.**
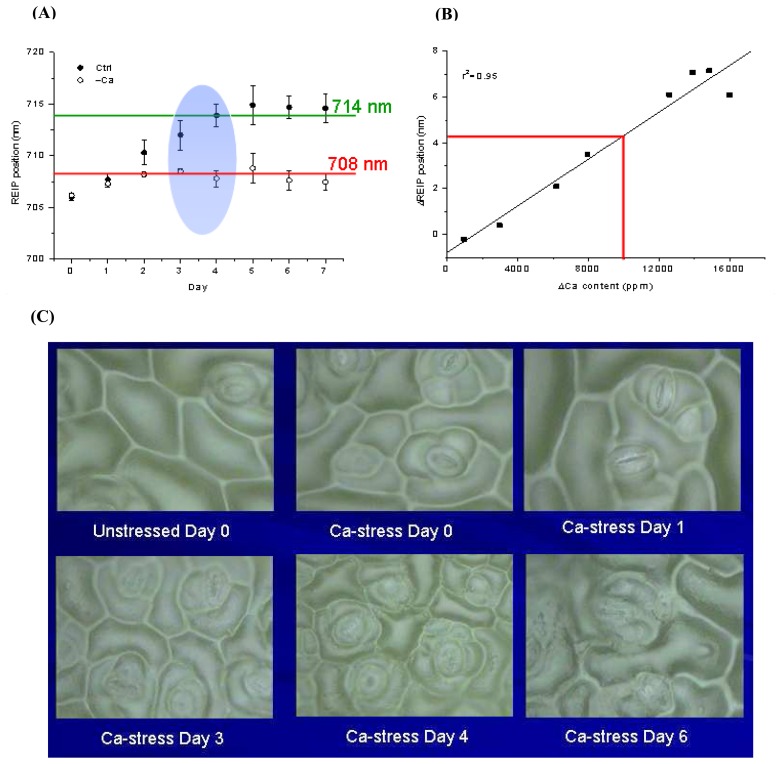
Spectral and morphological effects of calcium deprivation in *Brassica* sp. **(A)** REIP shifts in unstressed (Ctrl) and Ca-deprived (-Ca) plants as a function of time. The REIP position stabilizes around 714 nm in the nutrient-sufficient plants (green line) and 708 nm in Ca-deprived plants (red line). Significant deviation in REIP position between nutrient-sufficient and Ca-deprived plants from day 3-4 onwards (highlighted in the blue oval) coincides with obvious cellular breakdown shown in panel C. **(B)** Linear relationship between ΔREIP and ΔCa. The red line indicates the critical ΔREIP and ΔCa values above which plants are deduced to have entered into a deficiency state (adapted from Li et al. 2005 [78]). **(C)** Breakdown of cell structure in the abaxial epidermal surface with progression of calcium deprivation.

In our lab, we used a different combination of spectral features to tease out signature diagnostic information of mineral deficiencies in a model leafy plant, *Brassica chinensis* L. var *parachinensis* (Bailey) grown under hydroponics conditions. Leaf reflectance spectra (R) over the visible range from 380–780 nm were collected, and normalized inner reflectance (NR^I^) spectra were calculated to remove the effects of external reflectance and inner leaf scattering [95]. NR^I^ was then transformed into CIELAB color values, which simplified the whole visible spectrum into three values. We used REIP shifts and CIE L*, a* and b* values as composite predictors of specific elemental stresses. It was found that REIP shifts towards shorter wavelengths provided useful pre-visual cues for Ca deficiency in plants [78]. A linear relationship between the differences in the REIP (ΔREIP) and leaf Ca content (Δ[Ca]) of Ca-stressed and unstressed plants was found (r^2^ = 0.95). Significant deviations in red edge position and leaf Ca content were observed as early as three days after the imposition of calcium deprivation in young terminal leaves and these corroborated well with concomitant changes in the breakdown of cell structure on the abaxial epidermal surface (Figure 4). There were no significant differences in the ΔL*, Δa* and Δb* values between Ca-deficient and Ca-sufficient young plants [96].

In contrast, Fe-deprived plants entered into a deficiency state very rapidly. The direct effect of Fe on leaf chlorophyll content allowed CIELAB color values to be used for pre-visual detection of Fe deficiency 2 days before the appearance of visually distinguishable morphological changes [46]. Iron-stressed plants are characterized by a marked increase in L* (greater reflectance) and b* (more yellow) and no changes in a* values of young terminal leaves. Additionally, iron-deficient leaves also manifest red edge positions at shorter wavelengths compared with unstressed plants. Interestingly, in our preliminary observations of Mg-deprived plants, apparent spectral changes occurred in leaves in mid-positions of the plant with an increase in a* value (movement away from green towards red) but no significant changes in L* and b* values. Although these latter results require further confirmation from larger scale trials, our observations are in agreement with the findings of Graeff et al. (2001) [97] who also reported a significant increase in a* values in Mg-deficient maize plants compared to nutrient-sufficient plants. Hence, the possibility of establishing unique spectral signatures for each elemental deficiency is promising. While the classical morphological symptom of Fe and Mg deprivation is chlorosis, the CIE system indicates that the manner of color change can be distinguishable where Fe-deprived leaves become ‘more yellow’ while Mg-deprived leaves become ‘less green’. This subtle color variation may be attributed to the different impact that each elemental deficiency has on the level of the various types of photosynthetic pigments in the leaf. Fe deficiency leads to more severe decline in chlorophylls compared with xanthophylls (lutein and xanthophyll cycle pigments) and hence may explain the more ‘yellowish’ nature of Fe-deprived leaves [98-99]. The spatial position of the leaf on the plant where spectral changes are first detected also forms an added level of discrimination on the basis of the mobility of essential nutrient elements in the plant.

### 2.4 Opportunities for developing non-contact / remote sensing

There are four main remote sensing techniques classified according to the operating spectral regions: optical, thermal, radar and LIDAR. Of these, optical remote sensing, which relies on reflection of sunlight in the visible, near and middle infrared regions (400 – 2500 nm) by target objects, is the most well established approach for vegetation mapping. Remote sensing approaches that provide high spatial resolution data has primary applications in managing forest inventory related to assessing stock levels and classification of vegetation types [100-101]. Systems that provide high spectral resolution data permit mapping of vegetation condition associated with health and nutrition, and biological invasions (pests, diseases and weeds) [102-103]. A wide range of sensors from field-based instruments extending to airborne and satellite imaging spectrometers have been established, providing spatial information at very coarse scale to very high 1 m resolutions [100, 104-105]. Where the requirement is to obtain high spatial resolution spectral data at frequent intervals and low cost, imaging spectrometers may be mounted on tractors or other mobile farm equipment for ground-based measurements. To achieve wider area coverage, sensors can be mounted on aircrafts. Airborne imagers include CASI (Compact Airborne Spectrographic Imager), AVIRIS (Airborne Visible Infrared Imaging Spectrometer) and HyMap that are capable of providing hyperspectral data with wavelength coverage from 400 – 2500 nm. Experimental spaceborne imaging spectrometers mounted on-board a space craft or satellite provide advantages of largest area coverage at relatively lower cost per unit area. High spectral and radiometric resolutions can be achieved from Hyperion on the EO-1 satellite launched by NASA in 2000 and from the IKONOS satellite launched in 1999.

Applied to vegetation mapping, the sensors described above are driven primarily by leaf reflectance properties and canopy structure. However, extending current understanding of light interaction at the leaf level to field scale for assessing plant physiological performance at canopy level by remote sensing is a challenging one. This is because the overall shape of canopy reflectance spectra is influenced not only by processes operating at leaf level but also by variations in soil background reflectance, light scattering by surrounding objects, 3-D canopy and under-storey architecture and atmospheric conditions [106]. A further significant problem for detection at canopy level is the presence of strong absorption by water in the middle infrared regions that can mask small reflectance changes attributed to deviations in plant status. Nevertheless, remote monitoring of plant health by reflectance spectroscopy in the visible and near infrared waveband regions is achievable. Simple ratioing techniques have allowed detection of water stress in the classical NIR waveband regions while metal toxicity and deficiency conditions were detected by notable changes in the visible light range despite high variability in data and background noise [107]. However, such simple techniques do not allow for discriminatory identification of the specific stress in question. Current optical remote sensing techniques are not capable of isolating element-specific deficiency-induced spectral changes: it can only provide indication of the presence of stress but not identify the stressor [5, 108]. Hence, the identified problem areas will require further field analysis and survey. In practice, instruments with high spectral resolution (bandwidth <10nm) that permit more detailed analyses are required for remote detection of subtle deviations in spectral features that may be too narrow to be discriminated by coarse spectral resolution instruments [14, 109-110]. Successful application of the various remote sensing techniques must continue to be driven by improved understanding of the relationships between vegetation biochemical and structural factors on light reflectance and backscatter at canopy level.

Future developments would require remote fluorescence sensing capabilities with advances in active light sources and canopy level / airborne imaging platforms at high spatial and spectral resolution. Laser-induced fluorescence spectroscopy (LIFS) and laser-induced fluorescence imaging (LIFI) offer opportunities for measurement of signal yields over several orders of magnitude, in contrast with the more limited signal strength obtained with conventional reflectance measurements. It is possible that fluorescence spectra can complement passive reflectance measurements to yield signature physiological information on plant health. Scientists at the US Department of Energy Special Laboratories Technology combined LIFS and LIFI for monitoring plant responses to heavy metal uptake in surface contaminated soils [111]. LIFI affords recording of spatial data while LIFS records fluorescence spectra of a single point. Progressive advances in imaging techniques of whole leaves or canopies rather than point data measurements would provide more representative information with greater resolution and sensitivity for early stress detection [36]. Emerging technologies that provide information on plant biochemical and anatomical status, that move investigations from point measurements of single leaves to imaging of individual plants through to canopy, field and regional scales and to 3-D LIDAR imaging, open up exciting possibilities for a diversity of applications [112-116].

## 3. Transgenic approaches for sensing plant stresses

### 3.1 Biosensing organisms

The conventional approach for stress detection and monitoring described in previous sections hinges upon teasing out signature cues from deviations in thermal, fluorescence and/or reflectance characteristics. In many cases, however, changes in spectral patterns were non-discriminatory and hence, identification of the specific stressor is not possible. Transgenic reporter organisms or biosensors provide a viable alternative approach to stress detection. These organisms are genetically engineered to elicit an inducible biological signature with user defined characteristics that are distinguishable from endogenous background signals [117-118]. When combined with the traditional sensor systems, this strategy allows for concomitant signal creation in response to specific stresses, signal detection and exploitation.

There are essentially three classes of biosensing organisms: general, semi-specific and specific biosensors [119-120]. In general transgenic biosensors, the reporter genes are driven by a constitutive promoter and hence are eliciting signal outputs constantly. The semi-specific and specific biosensors drive reporter gene activity under inducible promoters. The difference between the two is that the latter is activated by a single or narrow range of inducers or stress factors while the former is triggered by a broader range of compounds or conditions. Sometimes, a regulatory protein may be involved to activate or repress the promoter

The more established biosensing organisms are based on bacterial bioreporters that have been established for environmental sensing of a wide array of target compounds and biological parameters [121-123]. These organisms have been engineered to emit optical signals in response to aromatic hydrocarbon, heavy metal, TNT or pathogen-induced stresses. These biosensors are often regulated at the gene transcription level by promoters and transcription factors that are responsive to the target compounds or conditions of interest. Subsequently, analysis of the overall reporter response provides an optical feedback that can be measured as changes in fluorescence, absorbance or reflectance (or color) [121, 124].

There is also increasing interest to develop transgenic plant biosensors to provide in situ monitoring of biological, chemical or physical properties in its immediate environment [125]. Plant phytosensors are particularly advantageous in that they are environmentally innocuous, easily propagated on a large scale and dependent on renewable solar energy. Positioning of multiple phytosensors within the environment can provide high spatial resolution information. Essentially, the plant phytosensor constitutes the biological recognition and transducer element of a classical biosensor in eliciting a measurable signal response to a target compound or condition. To create an ideal plant sensor, the choice of the target responsive promoter fused to a suitable reporter gene should produce an inducible system with the following properties:

1. There should be no or at most weak basal (uninduced, background or leaky) reporter gene expression.
2. The fusion gene should be highly inducible.
3. The range of compounds or conditions that elicit gene expression may be broad or narrow; in other words, specificity is according to user defined objectives.
4. The intensity of the signal should be well correlated with the concentration of the inducer compound or the severity of the physical/biological condition.
5. The signal response should be easily measurable with a high dynamic range of inducer concentration or conditions.
6. The spatial distribution of the signal should be uniform throughout the plant or a specific tissue depending on the specific application.
7. The temporal response should be appropriate to user objectives; for example, for sensing water-deficit conditions in plants, a measurable response should be elicited at early onset of water stress before plant entry into permanent wilting point.
8. “Switch-off” of the signal should be possible once the inducer compound, triggering event or condition is removed.
9. The optical characteristic of the signal should be distinct from any interfering background noise within the plant environment to give high signal-to-noise ratio.

Currently, no ideal phytosensor system meets all these criteria. There has been progress in discovering and characterizing a range of inducible plant promoters and reporter proteins that will support future development of phytosensor systems for a wide array of applications. These developments must also be supported by advances in imaging technologies that provide sensitive, accurate and quantitative measurements of reporter protein expression under the control of specific stress regulatory elements, not only in the laboratory but also in the field.

### 3.2 Inducible promoters for stress detection

Successful development of environmental and plant health phytosensing hinges on the identification of inducible promoters involved in plant stress responses and chemical regulation so that control of the desired gene expression pattern can be achieved. A range of promoters that are inducible by stress (salinity, flooding, drought, temperature, heat shock, wounding due to pathogen or insect attack), nutrients (nitrate and copper-inducible), growth regulators (abscisic acid) and chemicals (tetracycline and insecticide) have been described [126-127]. Further studies are needed to understand the complex regulatory networks associated with stress responses, define their specificity and continue with promoter discovery so that the utility of these regulatory DNA for wide sensing applications in the field can become a reality.

The conventional method to identify plant promoter elements relies on serial deletions of a potential promoter that is fused to a reporter gene followed by gain- and loss-of-function analyses. By analyzing the expression profile of the deletion mutants in a transformed cell, critical portions of the promoter that is essential and sufficient to control transcription can be determined [128]. The current trend is towards using more robust and high throughput array-based expression profiling techniques for identifying stress responsive genes [129-133]. Klok et al. (2002) [134] exposed *Arabidopsis* to anaerobic stress conditions to mimic flood and waterlogging of plants which deprive roots of oxygen. They were able to use micro-array analysis to study changes in the *Arabidopsis* gene expression and identify genes, for example, signal transduction components that are transcriptionally responsive to the low-oxygen treatment. Subsequently, they discovered new promoter elements from over-expressed promoters of the anaerobic responsive genes. Molecular discovery and identification are also augmented by powerful biocomputational tools; the classical alignment based motif-discovery algorithms like MEME and Gibbs [135-136], expression profiling of clustering genes (hypothesizing that transcription will be regulated by the same transcription factors) and phylogenetic footprinting (identifying conserved areas in known promoter sequences of several orthologous genes from closely related organisms) [137-140]. With the elucidation of a variety of stress response mechanisms such as antioxidation, heat-shock responses, nutrient-starvation and membrane damage response, it is possible to create a versatile array of biosensors to a selection of analytes by linking the DNA promoter elements of stress response proteins to available reporter genes.

### 3.3 Reporters

The few reporter genes that are commonly used as reporters of biosensors are the GFP gene from *Aequorea victoria*, luciferase (luc) gene from the firefly or lacZ gene from *Escherichia coli* [141]. An ideal bioreporter candidate would be one that expresses a well-detectable signal that is non-toxic to the host, sensitive, fast, requires no substrate involvement and allows for non-invasive *in vivo* visualization of intracellular molecular events. The level of reporter protein expression should be correlated with the exposure time, the target compound concentration or stress time. It should be quantifiable on the basis of its abundance (eg: GFP or red fluorescent protein) or by measuring its activity (eg: firefly luciferase). These features are critical for the utility of reporters as indicators of the severity of chemical, physical and biological stresses [142].

Of the many fluorescent reporter proteins, green fluorescent protein (GFP) and its mutants are probably most widely used [143-145]. They come in various colors that are altered with improved stability and enhanced signal intensity. Most importantly, they do not need substrates or ATP to fluoresce and can now be targeted to specific sites such as in the chloroplasts or cytoplasm in plants to track spatial and temporal stress induced or repressed gene response to stimulus. Another good feature of a candidate bioreporter is the ability for multiple labeling of different plant parts. As each fluorescent protein has its own characteristic excitation and emission spectra, different fluorescent protein colors can be distinguished with the appropriate optical filter sets. Recently developed fluorescent proteins, Keima and its variants, have been designed with a large Stokes shift that can be simultaneously imaged in different emission colors with a single wavelength excitation at 440 nm [146]. Kaede has a photoactivation characteristic where its green chromophore is convertible from green to red upon excitation by UV or violet light of 350 – 400 nm [147]. This fluorescent protein is useful for tracking dynamic changes upon sensing external stimuli. Another fluorescent protein, Dronpa is also reversibly convertible between bright and dark states upon photoactivation by light around 390 – 405 nm [148-149].

Sensitivity is essential for a bioreporter to be efficient as an *in vivo* reporter system. In gene expression studies, a strong and quick responsive reporter protein is important for the quantitative measurement of gene expression levels. With half-life of more than 24 hours for some very stable reporter proteins, it may accumulate in targeted sites at low expression level or even when in the uninduced state. This may be undesirable for some studies as it can lead to high basal readings, mask changes in expression levels or hamper measurements of temporary target signal fluctuations. Hence, shorter half-life reporters have been created to prevent its over-accumulation, allowing for a faster response time and higher induction signals which increased the bioreporter sensitivity tremendously [150-151].

Luminescent proteins are also frequently used as reporters of gene activity. It gives lower background interfering signals and they do not require external light excitation. Their primary disadvantage is that they require co-substrates and energy such as ATP from living cell metabolism. Well known examples of luminescent proteins are *Renilla reniformis* luciferase, bacterial luciferase and firefly luciferase [152-153].

### 3.4 Transgenic phytosensors

Plants are generally equipped with defense mechanisms to avoid or reduce damage from both biotic and abiotic stresses. Prompt response to stress is attributed in part to selective translation of pre-made mRNAs and the activation of inactive transcription factors like bZIP proteins. It is known that stress responses vary between species of plants and the types of stress. There is increasing evidence that defense responses to biotic and environmental stresses are linked and some common response genes associated with pathogen attack and physical stresses have been identified [154]. The general activation of these genes as a result of overlapping signaling pathways thus limits their utility to drive specific reporter expression in transgenic plant sensors. To date, there are few reports on field-ready transgenic phytosensors and most of these applications are still in the development stage. There are reports on genetically engineered signals triggered in response to nutrient starvation, environmental contaminants, pathogen attack and drought stress.

Hammond et al. (2003) [155] used a cDNA microarray approach to identify genes that are responsive to phosphorus starvation in *Arabidopsis*. However, many of the upregulated genes after P withdrawal were also known to be induced by other stress factors including wounding, pathogen attack, salinity, drought, oxygen deprivation, heat shock and cold. To develop plants for sensing P status in plants, identification of genes that are significantly upregulated at the early onset of P-induced stress before plant growth is adversely affected are required. The upregulation of these genes should also be sustained while plants are in the P-deprived state and responsive only to P deprivation and not to any other elemental deficiency or environmental challenges. Hammond and coworkers found 18 such genes that were upregulated between 28 – 100 hours after P withdrawal and impartial to other stress stimuli. The cellular functions of most of these genes were associated with metabolism and one was a transcription factor. A proof-of-concept DNA construct comprising the promoter element of *SQD1*, a gene involved in sulfolipid biosynthesis, fused to a GUS marker gene successfully demonstrated reporter β-glucuronidase activity in *Arabidopsis* shoots 20 h after P withdrawal and a steady increase in activity up to 220 h.

An innovative approach was developed by Kovalchuk and coworkers who devised plant recombination and mutation assays for rapid and precise detection of radioactive and heavy metal contamination in soils [156-157]. Transgenic *Arabidopsis* plants carrying two overlapping truncated versions of the β-glucuronidase (GUS) marker gene were regenerated. Unless homologous recombination occurs at the transgenic locus to allow production of the functional enzyme, all plant tissues will appear white instead of blue when treated with GUS substrates. Because the increase in the frequency of homologous recombination events is dose-dependent to the radiation levels, restoration of GUS activity visualized by a blue coloration provides a facile and visual means of monitoring radioactive contamination in the field. In subsequent work, an additional mutation assay was also developed where the GUS gene was designed in such a way that translation of an active enzyme takes place only when a spontaneous mutation occurs. Both the GUS-based recombination and mutation transgenic systems were then applied to monitor soil contamination by heavy metals, Cd, Pb, Ni, Zn, Cu and As_2_O_3_. Recombination and mutation events occurred at a higher frequency when plants undergo heavy metal stress resulting in translation of a functional GUS enzyme that can be visualized by a color change.

In a recent study, the green fluorescent protein gene under the control of a pathogen-inducible promoter was successfully triggered at the early stages of disease development in transgenic tobacco [158]. By transforming *Nicotiana tabacum* (tobacco) with a construct containing GFP as a reporter gene linked to a promoter of *gn1*, a tobacco β-1,3-glucanase gene, a transgenic biosensor was created for early disease diagnosis. The *gn1* promoter was shown to be sensitive to both fungal pathogen *Plectosporium tabacinum* and salicylic acid that is synthesized during pathogen attack. Under the control of this pathogen-inducible promoter, GFP transcripts and its proteins were found to be accumulated in the roots and older leaves. However, detection of GFP fluorescence in this study was successful using a fluorescence microscope but not fluorescence spectroscopy which confines the utility of this system to the laboratory.

In our laboratory, we have successfully developed a dual component system comprising an optical detector and novel transgenic indicator plants that emit green fluorescence signals for sensing early onset of drought stress [159]. The rationale for our work is that efficient use of applied water in agriculture requires development of strategies for site-specific irrigation on a needs base. Uniform irrigation application is not only economically untenable but also environmentally unsustainable. The existing strategies for determining crop water requirement has relied on a combination of information on climatic conditions, spatial mapping of soil properties, irrigation rates together with direct plant measurements of water status like water potential using a pressure chamber, stomatal conductance by porometry and canopy temperature by infrared thermography. These methods are unwieldy, require destructive sampling and difficult to translate for field use. To this end, the capability of monitoring plant water status non-invasively and in real-time will aid technologies towards targeted water use both spatially and temporally.

**Figure 5.**
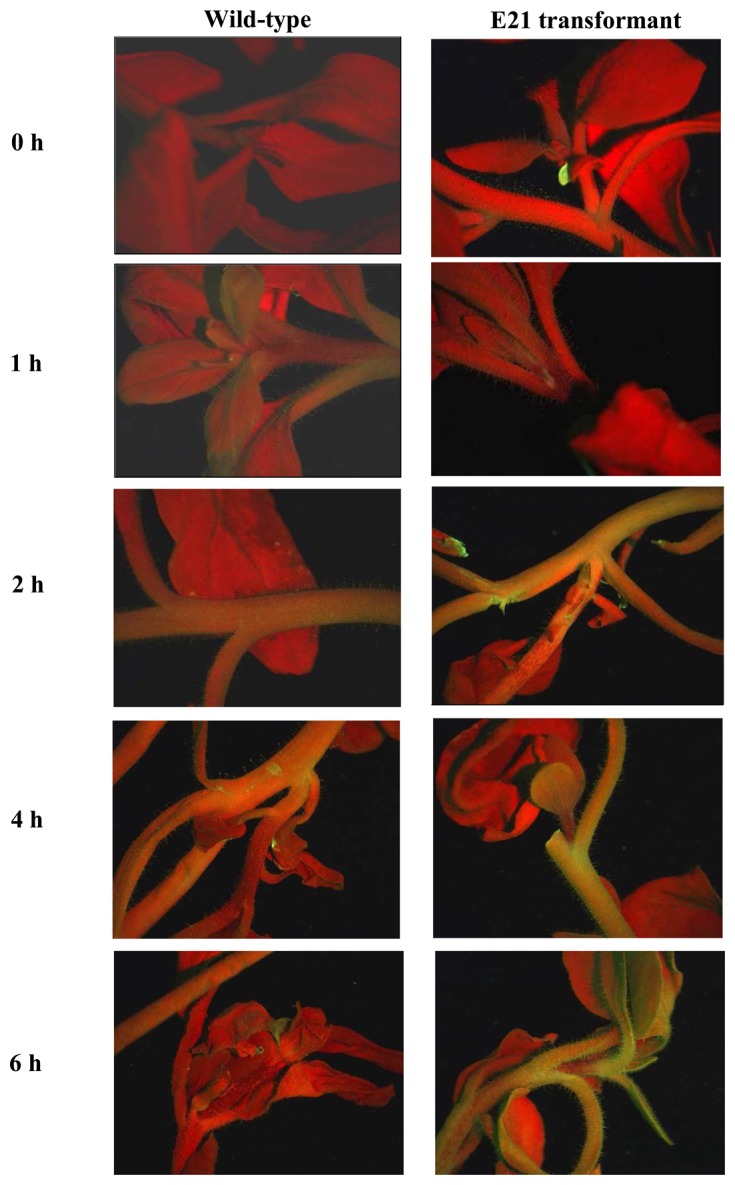
Fluorescence stereoscopic microscope images of wild-type and E21 transformed plants at 0 h, 1 h, 2 h, 4 h and 6 h dehydration time points, showing increasing EGFP expression in stems, petioles and terminal leaves with progression of water stress (reproduced from Chong et al. 2007 [159]).

**Figure 6.**
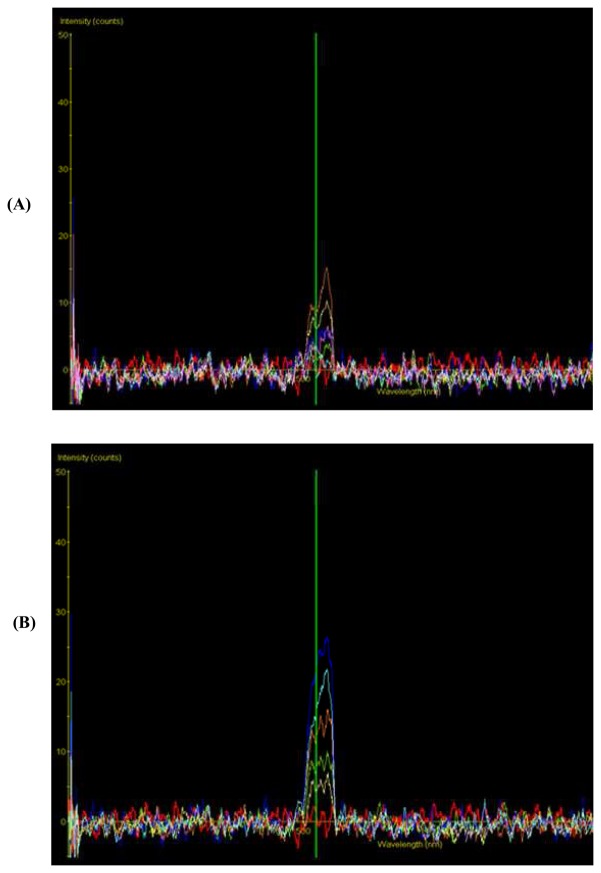
Spectroscopic detection of EGFP emission from (A) wild-type and (B) E21 transformant at 0, 1, 2, 4 and 6 h time points following onset of desiccation stress. The Y-axis represents fluorescence intensity (arbitrary units) and the X-axis represents wavelength from 380 nm to 850 nm. The expected EGFP emission peak at the 509 nm position is indicated by the green vertical line (reproduced from Chong et al. 2007 [159]).

Our plant phytosensor comprised transgenic *Petunia hybrida* harboring an *Arabidopsis thaliana* drought-responsive promoter linked to the enhanced green fluorescent protein (EGFP) gene. There are many genes known to be responsive to drought stress and these include those specifying membrane-associated proteins, solute transporters, reactive oxygen scavengers and thiol proteases [160-161]. We chose to isolate the promoter element of the *rd21A* gene that encodes a drought-responsive cysteine proteinase to generate our bioreporting fusion gene construct. These regulatory elements have been shown to be unresponsive to cold or heat stress nor transcriptionally induced by the plant hormones, abscisic acid (ABA) and gibberellin [162]. However, the *rd21A* gene was highly responsive and transcriptionally active during drought and severe salinity stress. The detection of EGFP emission was via a fluorescence stereoscopic microscope while quantitation of signal intensity was achieved through coupling a spectrometer to the detection port of the microscope. Visible EGFP expression was observed 2h after exposing the plants to air dehydration with increasing signal intensity up to the 6h time point where the plants entered the stage of irreversible damage (Figure 5). The emission spectra obtained from the dehydration-stressed transgenic *Petunia* was broad (500 – 520 nm) and had a peak emission at 516 nm instead of the expected 509 nm (Figure 6). Red-shifting of the EGFP emission peak was attributed to signal distortion due to the optical configuration of the microscope and overlapping background noise from endogenous green autofluorescence contributed possibly by plant constituents like lignin and flavins [163-164]. Weak induction of EGFP in response to water stress warrants further optimization of the methodology for spectroscopic signal quantification.

Currently, although the *rd21A* promoter performed adequately from a temporal perspective, non-uniform spatial expression of EGFP in the plant, EGFP chromophore maturation behaviour and high endogenous green autofluorescence in *Petunia* present considerable challenges to translate this system for field use. Non-uniform spatial expression of GFP even under the regulation of constitutive promoters, like the 35S promoter, has also been observed by other workers [165-167]. Thus, the requirements for technology exploitation of transgenic plant sensors are to increase the spatial uniformity, signal intensity and specificity of reporter protein expression.

In most studies, instrumentation for *in vivo* detection of GFP fluorescence emission has primarily been achieved via fluorescence/confocal laser microscopy and fluorescence imaging spectroscopy [168-169]. However, these approaches are limited to laboratory settings and do not lend themselves to field use. In addition, the need for destructive sampling for image analysis also becomes an impediment for continuous real-time monitoring. Progress in instrumentation development for in-field GFP measurements from whole plants is palpable. For example, a handheld fibre optic fluorometer commercialized as the GFP Meter (Opti-Sciences Inc, USA) can be used to detect fluorescence signals directly off a whole leaf [170]. In addition, we have previously reported and improved on a portable fibre optic spectroscopic system that allows non-destructive *in planta* detection of EGFP emission signals in tobacco [171-172]. However, the system is limited to single point data collection that requires contact between the probe and leaf surface. Further modifications to the probe head are underway to allow signal detection without interference from ambient light so that field data can be collected in the day time. Currently, progress made in developing instrumentation for remote fluorescence sensing is promising and will continue to require greater understanding of signature pattern creation and responses from more robust ground-based measurements in the near term [173].

## 4. Space Applications

The development of bioregenerative life-support systems that encompasses automated components for sensing, monitoring and controlling plant growth in space has been a research priority in the National Aeronautics and Space Administration's (NASA's) strategic plans. This has been due to accumulating evidence showing the positive psychological benefits of human interaction with plants as well as the importance of supplying food that closely resembles that of earth to astronauts, especially on long term space missions [17]. In addition, bioregenerative systems are of great importance to provide continuous supply of life support components of fresh food, oxygen and clean water for the crew. This approach provides an attractive alternative to current practices involving costly stowage and resupply which becomes untenable particularly when the distance covered in space missions increases. However, the challenges for successful extrasterrestrial plant production lies in the limited understanding of the genetic, physiological and structural pertubations as well as adaptive mechanisms of plants under constraints of complex enclosed growth chambers and microgravity environments with limited light and oxygen supply [3].

Remote and non-invasive monitoring of crop growth and health status represent an initial step towards a systems approach for integration into a whole production system. Already some framework for controlled environment plant production system (CEPPS) operated under an automated and controlled environment concept (ACE) has been described by Giacomelli et al. (1994) [175] and Kitaya et al. (2000) [176]. Such integrated systems comprise 4 major components that are to be monitored and controlled; coordinates in time and space, machine and task status, plant growth and health status and environmental conditions in terms of temperature, nutrient delivery, water relations and light quality, intensity and photoperiod. Advances in the development of optical/chemical sensors and sensor arrays for environmental control and systems management has also been described [177-179]. Currently, these systems are not commonplace for open field cropping but some practical applications under controlled greenhouse environments are possible [180]. Model systems developed from terrestrial studies provide the framework for evolving integrated crop production systems in extraterrestrial environments.

Under NASA's Controlled Ecological Life Support System (CELSS), the strategy is to grow plants hydroponically under controlled environments where an intelligent monitoring system is required to track plant health status and early signs of stress. Optical sensors and their attributes are based on an understanding of the biological and physical parameters which firstly can be described and secondly linked to online feedback control systems for modulation of the plant growth system. Automation and remote monitoring of the plant parameters require the integration of input data acquisition and analysis with the hardware sensor. Manipulation, organization and interpretation of the data form the basis for diagnosis of plant health status and management decision making. Machine vision has been used to monitor plant health [181]. Images of the plants are captured continuously at predefined intervals throughout the growth cycle. Information on size, shape and color are extracted from these images and complex algorithms, statistical classifiers and neural networks that create and organize weighted connections between processing elements are developed to define plant health status. As such, characterization of the general growth patterns and color distribution of each specific plant in their healthy state is required and forms the reference basis by which deviations from normal development due to stress are determined and assessed. Color-oriented and volume-based classification forms the major components for analysis. Calibration strategies must account for variations in instrumentation, plant and environmental conditions at the point of data collection. Some key factors include light intensity, spectral characteristics of illumination source, leaf orientation with respect to light source and sensor, leaf distance from the sensor, degree of interference from ambient light, gain adjustments, focal length and field of view of camera.

## 5. Concluding remarks

The interaction of plants with its ever changing and heterogenous surroundings is dynamic and highly complex. In turn, the presence and activities of plants modulate the environment at the micro and macro levels. Understanding the physical and biological responses of plants to environmental stresses at the leaf level represents the first step to identifying unique spectral responses for extension to the canopy and regional scales. The road ahead to translate plant optical characteristics as stress predictors from the laboratory to the field or extraterrestrial environments is indeed challenging. There is much room for progress in instrumentation that allow higher resolution so that smaller variations in vegetation reflectance or signature fluorescence spectra can be detected from ground and aerial platforms, at local and regional scales. Multidisciplinary investigations must combine knowledge of sensor developments, environmental science, plant biology and computer programming. Research is needed to define species-specific spectral reflectance properties of normal or unstressed plants so that stress-related effects can then be distinguished and related to specific stressors. Another future challenge is to link the measurement system to automation for fine tuning or adjustments of setpoint plant growth conditions which must then trigger appropriate response(s) with respect to task execution. Hardware instrumentation must be complemented with powerful software analytical tools that encompass algorithms and databases to handle all imaginable plant health scenarios. Finally, innovative strategies are needed to allow categorization and representation of the milieu of practical situations encountered in the cropping cycle.
